# Draft Genome Sequence of *Staphylococcus aureus* S681, a Tetracycline-Sensitive Livestock-Associated Methicillin-Resistant Clonal Complex 398 Strain

**DOI:** 10.1128/genomeA.00805-17

**Published:** 2017-08-17

**Authors:** Marc J. A. Stevens, Roger Stephan, Sophia Johler

**Affiliations:** a Institute for Food Safety and Hygiene, Vetsuisse Faculty University of Zurich, Zurich, Switzerland; b Laboratory of Food Biotechnology, Institute of Food, Nutrition and Health, ETH Zurich, Zurich, Switzerland

## Abstract

We present the draft genome sequence of an atypical tetracycline-susceptible livestock-associated methicillin-resistant *Staphylococcus aureus* (MRSA) strain. It contains 2,817,340 bp and 2,858 coding sequences, including 6 rRNA operons, 56 tRNAs, and 4 noncoding RNA (ncRNA) genes. The strain harbors a *tet*(M) gene, but 15 point mutations in amino acids are present that likely impair the functionality of TetM.

## GENOME ANNOUNCEMENT

Methicillin-resistant *Staphylococcus aureus* (MRSA) is a leading cause of nosocomial infections and a major public health concern. Livestock-associated methicillin-resistant *Staphylococcus aureus* characteristically harbors not only *mecA*, conferring resistance to beta lactams, but also the tetracycline resistance determinant *tet*(M), alone or in combination with *tet*(K) and/or *tet*(L) (1, 2). In this study, we present the draft genome sequence of *S. aureus* S681, a tetracycline-susceptible livestock-associated MRSA. The strain was isolated from a pig carcass from a slaughterhouse in Switzerland and was shown to belong to *spa* type t034 and clonal complex 398 (CC398) (3). The strain exhibited resistance to ampicillin, cefoxitin, oxacillin, and penicillin in a disk diffusion assay. While the strain showed a tetracycline-susceptible phenotype, a DNA microarray assay yielded a positive hybridization result for a probe targeting *tet*(M) (4).

In this study, the genome of strain S681 was sequenced using Ilumina Miseq 125-bp paired-end sequence technology as described previously (5). The raw reads were paired in CLC genomics workbench 9.0, resulting in 1,560,556 reads with a mean size of 250 bp. The reads were assembled in CLC genomics workbench 9.0 using standard settings, including scaffolding. The assembly was corrected by NCBI PGAP. The final version contains 30 contigs with sizes between 1.5 and 440 kbp. The average read coverage of the genome was 120-fold.

The genome of *S. aureus* S681 contains 2,817,340 bp and 2,858 coding sequences, including 6 rRNA operons, 56 tRNAs, and 4 noncoding RNA (ncRNA) genes. The G+C content of the genome is 32.3%.

Remarkably, the tetracycline resistance gene *tet*(M) (locus CDQ86_05625) was detected in the genome of this phenotypically tetracycline-susceptible strain. However, 15 point mutations in amino acids were present, which quite likely impair the functionality of TetM.

### Accession number(s).

This whole-genome shotgun project has been deposited at DDBJ/ENA/GenBank under the accession number NISC00000000. The version described in this paper is version NISC01000000.
